# Maternal B vitamins: effects on offspring weight and DNA methylation at genomically imprinted domains

**DOI:** 10.1186/s13148-016-0174-9

**Published:** 2016-01-22

**Authors:** Lauren E. McCullough, Erline E. Miller, Michelle A. Mendez, Amy P. Murtha, Susan K. Murphy, Cathrine Hoyo

**Affiliations:** Department of Epidemiology, University of North Carolina Chapel Hill, Chapel Hill, NC USA; Lineberger Comprehensive Cancer Center, University of North Carolina Chapel Hill, Chapel Hill, NC USA; Department of Nutrition, University of North Carolina Chapel Hill, Chapel Hill, NC USA; Department of Obstetrics and Gynecology, Duke University School of Medicine, Durham, NC USA; Department of Biological Sciences, North Carolina State University, Raleigh, NC USA; Rollins School of Public Health, Emory University, 1518 Clifton Rd NE, CNR 3037, Atlanta, GA 30322 USA

**Keywords:** B vitamins, Birth weight, Childhood weight gain, DNA methylation, Imprinted genes, Epidemiology

## Abstract

**Background:**

Inadequate maternal nutrition during early fetal development can create permanent alterations in the offspring, leading to poor health outcomes. While nutrients involved in one-carbon cycle metabolism are important to fetal growth, associations with specific nutrients remain inconsistent. This study estimates associations between maternal vitamins B_12_, B_6_ (pyridoxal phosphate [PLP] and 4-pyridoxic acid [PA]), and homocysteine (Hcy) concentrations, offspring weight (birth weight and 3-year weight gain), and DNA methylation at four differentially methylated regions (DMRs) known to be involved in fetal growth and development (*H19*, *MEG3*, *SGCE/PEG10*, and *PLAGL1*).

**Methods:**

Study participants (*n* = 496) with biomarker and birth weight data were enrolled as part of the Newborn Epigenetics STudy. Weight gain data were available for 273 offspring. Among 484 mother-infant pairs, DNA methylation at regulatory sequences of genomically imprinted genes was measured in umbilical cord blood DNA using bisulfite pyrosequencing. We used generalized linear models to estimate associations.

**Results:**

Multivariate adjusted regression models revealed an inverse association between maternal Hcy concentration and male birth weight (*β* = −210.40, standard error (SE) = 102.08, *p* = 0.04). The offspring of the mothers in the highest quartile of B_12_ experienced lower weight gain between birth and 3 years compared to the offspring of the mothers in the lowest (*β* = −2203.03, SE = 722.49, *p* = 0.003). Conversely, maternal PLP was associated with higher weight gain in males; higher maternal PLP concentrations were also associated with offspring DNA methylation levels at the *MEG3* DMR (*p* < 0.01).

**Conclusions:**

While maternal concentrations of B_12_, B_6_, and Hcy do not associate with birth weight overall, they may play an important role in 3-year weight gain. This is the first study to report an association between maternal PLP and methylation at the *MEG3* DMR which may be an important epigenetic tag for maternal B vitamin adequacy.

**Electronic supplementary material:**

The online version of this article (doi:10.1186/s13148-016-0174-9) contains supplementary material, which is available to authorized users.

## Background

Size at birth is a strong predictor of infant growth and survival and has been linked to lifelong health outcomes [1]. Low birth weight (LBW, <2500 g) is associated with augmented risk of adult chronic conditions including the following: obesity, cardiovascular disease, type 2 diabetes, and some cancers [2, 3]. The period of intrauterine growth may therefore be characterized by heightened vulnerability from both endogenous and exogenous factors.

While maternal nutrition during fetal development has been shown to have long-term health consequences for the offspring [4], the impact of specific nutrients on fetal and childhood outcomes remains unresolved. Nutrients involved in one-carbon cycle (1-CC) metabolism are essential for nucleic acid synthesis, DNA methylation, and cellular growth [5]. These nutrients are particularly important in fetal tissue differentiation. Thus, deficiencies in this pathway could be associated with fetal growth restriction, LBW, and early-life weight gain (WG). Understanding the association between these nutrients and offspring weight may be crucial in uncovering modifiable ways to prevent LBW and downstream health effects.

The 1-CC metabolism pathway provides methyl groups for DNA methylation reactions and is dependent on several enzymes in the presence of dietary micronutrients. Key micronutrients in this pathway include folate, choline, and co-factors such as vitamins B_6_ and B_12_. While the literature supports an association between low maternal blood folate concentrations and small size at birth [6], data regarding associations between fetal growth and maternal levels of vitamins B_6_, B_12_, and homocysteine (a marker of impaired folate status) remain equivocal [7]. Further, data linking maternal micronutrient concentrations to early-life outcomes are limited [8].

Inadequate maternal 1-CC nutrients have been linked to lower DNA methylation at the agouti locus [9] during critical periods of early embryonic development, and in this model epigenetic programs, the offspring to adult obesity, diabetes, and cancer [10]. The plasticity of the epigenome may offer a mechanistic link between maternal nutrition and adult disease [11]. DNA methylation is the most widely studied epigenetic modification and is integral in regulating gene expression [12]. Despite the importance of 1-CC nutrients in DNA methylation, few studies have investigated whether maternal micronutrient concentrations influence offspring DNA methylation patterns [13–15], and most have primarily focused on folate.

The aim of this study was to examine the association between maternal serum concentrations of vitamin B_12_ (B_12_), pyridoxal phosphate (PLP), 4-pyridoxic acid (PA), homocysteine (Hcy), and offspring weight at birth through age 3 years in an ethnically diverse population. We further sought to explore the potential effects of maternal micronutrient concentrations on the methylation patterns of genomically imprinted gene differentially methylated regions (DMRs). While there are more than 80 recognized imprinted genes in humans [16], we selected four DMRs (*H19*, *MEG3*, *SGCE/PEG10*, and *PLAGL1*) known to be important in fetal growth and development [17, 18] and previously associated with maternal B vitamin concentrations in cross-sectional studies [13–15].

## Results

The majority of women included in these analyses were aged 20–29 years (56 %), were black (42 %), and had low income (55 %). Notably, 26 % of the study participants smoked prior to or during pregnancy, and a large proportion of women supplemented with folic acid (FA) (89 %). Blood draw occurred during the first trimester for most participants (64 %). Table 1 provides the median (range) for maternal micronutrient concentrations of B_12_, PLP, PA, and Hcy by maternal and offspring characteristics. Concentrations of PLP and PA were substantially (*p* < 0.05) lower among Black than White mothers.

**Table 1:** Study participant characteristics by maternal B vitamin concentrations: Newborn Epigenetic STudy (*N* = 496)

Variable		B_12_ (ng/L)	PLP (nM/L)	PA (nM/L)	Hcy (umoL/L)
		*N* = 489	*N* = 496	*N* = 496	*N* = 480
	*N* (%)	Median (range)			
Age at delivery (year)					
20–29	278 (56%)	428.44 (56.13–2628.11)	6.66 (0.00–54.90)	2.85 (0.00–179.19)	0.69 (0.30–1.34)
18–< 20	22 (4%)	419.05 (216.96–834.27)	7.30 (1.25–32.19)	3.43 (0.00–24.92)	0.75 (0.44–0.98)
30–35	147 (30%)	495.38 (151.24–3664.91)	8.46 (0.00–173.86)	3.70 (0.00–218.44)	0.70 (0.33–1.54)
36+	50 (10%)	411.46 (148.62–2779.17)	9.02 (0.41–76.90)	2.85 (0.00–122.61)	0.71 (0.35–1.07)
Race/ethnicity					
Black	186 (42%)	431.76 (109.44–1249.75)	5.62 (0.00–34.58)	2.41 (0.00–130.60)	0.70 (0.30–1.54)
White	133 (30%)	493.67 (56.13–3664.91)	8.87 (0.00–173.86)	4.85 (0.00–218.44)	0.73 (0.44–1.19)
Hispanic	121 (28%)	432.25 (93.47–2779.17)	8.07 (1.60–30.24)	2.94 (0.00–30.01)	0.69 (0.33–1.22)
Preterm birth					
Term	468 (94%)	444.02 (56.13–3664.91)	7.35 (0.00–173.86)	3.21 (0.00–218.44)	0.70 (0.30–1.54)
Preterm	29 (6%)	485.71 (162.05–891.89)	7.91 (0.00–34.58)	3.24 (0.00–30.01)	0.66 (0.33–1.08)
Marital status					
Married	186 (38%)	485.98 (148.62–3664.91)	8.89 (0.00–173.86)	4.25 (0.00–218.44)	0.71 (0.30–1.22)
Never married	141 (29%)	443.03 (56.13–2628.11)	5.86 (0.95–34.58)	2.57 (0.00–130.60)	0.70 (0.35–1.34)
Living with partner	136 (28%)	406.87 (133.52–1570.18)	7.73 (0.00–31.59)	2.94 (0.00–35.09)	0.69 (0.44–1.54)
Other	28 (5%)	398.20 (128.05–2779.17)	7.55 (0.00–35.12)	2.55 (0.00–11.38)	0.71 (0.33–1.10)
Parity (at enrollment)					
Multiparous	320 (65%)	443.89 (56.13–3664.91)	6.74 (0.00–173.86)	2.77 (0.00–218.44)	0.69 (0.33–1.54)
Nulliparous	176 (35%)	454.01 (109.44–1600.50)	9.02 (0.00–63.59)	4.34 (0.00–186.65)	0.71 (0.30–1.21)
Household income					
<$25,000	215 (55%)	414.91 (128.05–2779.17)	6.66 (0.00–56.00)	2.71 (0.00–30.01)	0.68 (0.35–1.34)
$25,000–$49,999	54 (14%)	426.39 (162.05–855.61)	6.25 (0.00–173.86)	3.20 (0.00–218.44)	0.72 (0.30–1.54)
$50,000–$100,000	73 (18%)	500.13 (143.07–3664.91)	9.92 (0.00–76.90)	4.67 (0.00–179.19)	0.73 (0.43–1.19)
>$100,000	50 (13%)	507.86 (148.62–1600.50)	11.32 (1.34–63.59)	5.52 (0.79–186.65)	0.68 (0.44–1.10)
Education					
College graduate	145 (29%)	508.50 (148.62–3664.91)	9.15 (0.00–173.86)	4.60 (0.00–218.44)	0.70 (0.30–1.21)
High school/GED	108 (22%)	381.84 (56.13–1038.75)	6.02 (0.00–54.90)	2.63 (0.00–179.19)	0.70 (0.40–1.14)
Less than high school	166 (34%)	437.94 (93.47–2779.17)	6.78 (0.00–35.12)	2.74 (0.00–30.01)	0.68 (0.33–1.34)
Some college	75 (15%)	415.24 (195.26–1600.50)	7.08 (0.00–56.00)	3.18 (0.00–119.67)	0.72 (0.48–1.54)
Body Mass Index (kg/m^2^)					
18.5–24.99	195 (41%)	482.78 (93.47–2779.17)	8.35 (0.00–173.86)	3.95 (0.00–218.44)	0.70 (0.40–1.22)
25–29.99	149 (31%)	436.88 (140.80–3664.91)	6.92 (0.41–52.23)	2.75 (0.00–130.60)	0.69 (0.30–1.34)
>29.99	135 (28%)	392.29 (56.13–1570.18)	5.85 (0.00–39.70)	2.78 (0.00–36.57)	0.71 (0.40–1.54)
Smoking status					
No smoking	358 (74%)	450.11 (93.47–2779.17)	7.91 (0.00–173.86)	3.31 (0.00–218.44)	0.70 (0.30–1.34)
Smoking prior to pregnancy	49 (10%)	433.03 (195.26–3664.91)	7.71 (0.00–70.22)	3.42 (0.00–129.48)	0.65 (0.44–1.04)
Smoking during pregnancy	79 (16%)	447.62 (56.13–1570.18)	5.27 (0.35–39.70)	2.74 (0.30–130.60)	0.71 (0.40–1.54)
Folic acid supplementation					
Yes	433 (89%)	441.19 (56.13–2779.17)	7.04 (0.00–76.90)	3.08 (0.00–186.65)	0.70 (0.30–1.54)
No	52 (11%)	461.59 (143.07–3664.91)	9.87 (2.34–173.86)	3.49 (0.00–218.44)	0.69 (0.45–1.19)
Gestational diabetes					
No	461 (94%)	447.62 (56.13–3664.91)	7.35 (0.00–173.86)	3.10 (0.00–218.44)	0.69 (0.30–1.54)
Yes	30 (6%)	415.23 (195.26–841.23)	9.07 (1.43–33.29)	3.41 (0.93–36.57)	0.72 (0.53–1.07)
Gestational age at blood draw					
1st trimester	316 (64%)	455.71 (56.13–3664.91)	8.85 (0.00–173.86)	3.51 (0.00–218.44)	0.71 (0.30–1.54)
2nd trimester	166 (33%)	415.23 (109.44–2779.17)	5.77 (0.00–32.19)	2.70 (0.00–36.57)	0.66 (0.40–1.34)
3rd trimester	15 (3%)	301.80 (216.96–759.84)	3.06 (0.35–11.64)	2.16 (0.00–30.01)	0.62 (0.44–1.03)
Infant sex					
Male	248 (50%)	444.75 (109.44–3664.91)	7.33 (0.00–76.90)	3.25 (0.00–179.19)	0.70 (0.33–1.19)
Female	249 (50%)	447.77 (56.13–2779.17)	7.54 (0.00–173.86)	3.18 (0.00–218.44)	0.69 (0.30–1.54)

*B*_*12*_ vitamin B_12_, *PLP* pyridoxal phosphate, *PA* 4-pyridoxic acid, *Hcy* homocysteine

### Associations between maternal micronutrient concentrations (e.g., B_12_, PLP, PA, and Hcy) and birth weight

The mean birth weight in our study sample was 3294 g (standard deviation = 541 g). Table 2 presents adjusted regression coefficient estimates (*β*s) and standard errors (SE) for the multivariate association between maternal micronutrient concentrations and infant birth weight, as well as sex-specific estimates. All models adjusted for race/ethnicity, gestational age at blood draw and delivery, marital status, parity, income, pre-pregnancy BMI, and maternal smoking. While we observed a monotonic increase in birth weight with increasing PLP and decrease in birth weight with increasing Hcy among all participants, our estimates were imprecise.

**Table 2:** Adjusted regression coefficients for maternal B vitamins and birth weight by infant sex: Newborn Epigenetic STudy (*N* = 496)

		Infant sex	
	All participants (*N* = 496)	Male infants (*N* = 248)	Female infants (*N* = 248)
Maternal B vitamin	*β* coefficient, standard error, *p* value		
B_12_			
≤322.47 ng/L	Reference	Reference	Reference
322.48–446.04 ng/L	−12.89,70.83, 0.86	59.38, 104.12,0.58	−29.38, 101.01,0.77
446.05–575.51 ng/L	−35.89, 69.24, 0.60	−59.18, 105.57,0.58	13.55, 95.10, 0.88
>575.51 ng/L	1.85, 69.82, 0.98	108.19, 100.01,0.28	−63.02, 99.61,0.53
p for interaction		0.5276	
PLP			
≤3.76 nM/L	Reference	Reference	Reference
3.77–7.47 nM/L	−72.75, 71.50, 0.31	3.37, 106.39, 0.97	−126.43, 102.07, 0.22
7.48–12.05 nM/L	−45.61, 73.22, 0.53	−28.02, 114.15, 0.81	−15.02, 99.25, 0.88
>12.05 nM/L	39.81, 75.75, 0.60	43.48, 119.08,0.72	56.05, 100.69, 0.58
p for interaction		0.7014	
PA			
≤2.06 nM/L	Reference	Reference	Reference
2.07–3.21 nM/L	−13.80,70.48, 0.84	−50.67, 108.60, 0.64	49.43, 96.66, 0.61
3.22–5.93 nM/L	−35.02,70.51,0.62	−7.31, 107.61,0.95	−36.80, 95.43,0.70
>5.93 nM/L	9.95, 72.43, 0.89	16.03, 112.20, 0.89	35.43, 97.44, 0.72
p for interaction		0.9134	
Hcy			
≤4.40 umol/L	Reference	Reference	Reference
4.41–5.10 umol/L	−57.72, 70.94, 0.42	−143.95, 108.74, 0.19	27.65, 95.21, 0.77
5.11–6.00 umol/L	−72.86,71.21,0.31	−275.57, 105.70,0.01	76.91, 98.03, 0.43
>6.00 umol/L	−87.21, 70.48, 0.22	−210.40, 102.08, 0.04	27.33, 98.14, 0.78
p for interaction		0.1021	

Adjusted for gestational age at delivery, gestational age at blood draw, maternal pre-pregnancy body mass index, maternal race/ethnicity, parity, household income and maternal smoking

*B*_*12*_ vitamin B_12_, *PLP* pyridoxal phosphate, *PA* 4-pyridoxic acid, *Hcy* homocysteine

We found that the inverse association between Hcy and birth weight was most pronounced among male infants. The coefficient estimate among male infants in the highest quartile of maternal Hcy concentration was −210.40 (SE = 102.08, *p* = 0.04) while the corresponding estimate among female infants was 27.33 (SE = 98.14, *p =* 0.78). However, the cross-product term was not statistically significant (*p* = 0.10). Although we observed striking differences in the association between maternal B_6_ (PLP and PA) concentration and birth weight in infants born to women who reported FA supplementation (Additional file 1: Table S1), the estimates were imprecise due to the low proportion of non-supplementing women. These associations did not vary by race/ethnicity or maternal BMI, and exclusion of preterm infants did not substantially alter our findings.

### Associations between maternal micronutrient concentrations (e.g., B_12_, PLP, PA, and Hcy) and age 3 weight gain

Among all 273 children with anthropometric data at age 3 years, the mean WG was 11,741 g (standard deviation = 2250 g). Table 3 shows the linear associations between maternal micronutrient concentrations and age 3-year WG adjusted for gestational age at blood draw, maternal race/ethnicity, maternal pre-pregnancy BMI, household income, breastfeeding, and age 3-year caloric intake. The children of the mothers in the highest quartile of B_12_ concentrations experienced lower WG compared to the children of the mothers in the lowest quartile (*β* = −2203.03, SE = 722.49, *p* = 0.003). For maternal PLP concentrations, there was some indication of differences in association by offspring sex (*β*_males_ = 2943.29, SE = 1365.99, *p* = 0.04 and *β*_females_ = −1737.49, SE = 936.33, *p* = 0.08), but the differences were not significant at the 0.05 alpha level.

**Table 3:** Maternal B vitamins and 3-year weight gain by infant sex: Newborn Epigenetics STudy (*N* = 273)

		Infant sex	
	All participants (*N* = 273)	Male children (N-137)	Female children (*N* = 136)
Maternal B vitamin	*β* coefficient, standard error, *p* value		
B_12_			
≤322.47 ng/L	Reference	Reference	Reference
322.48–446.04 ng/L	−381.14, 732.87, 0.60	33.23, 1199.92, 0.98	−625.74, 891.01, 0.49
446.05–575.51 ng/L	−1395.75, 673.18, 0.04	−1010.30, 1086.31, 0.36	−1782.25,711.58, 0.02
>575.51 ng/L	−2203.03, 722.49, 0.003	−2154.17, 1224.31, 0.09	−1626.59, 837.17, 0.07
p for interaction		0.6998	
PLP			
≤3.76 nM/L	Reference	Reference	Reference
3.77–7.47 nM/L	448.65, 839.88, 0.60	1116.26, 1213.31,0.37	−1964.83, 1063.30, 0.08
7.48–12.05 nM/L	694.16, 897.50, 0.44	1220.60, 1530.15, 0.43	−1778.70, 1006.86, 0.09
>12.05 nM/L	1181.95, 866.33, 0.18	2943.29, 1365.99, 0.04	−1737.49, 936.33, 0.08
p for interaction		0.1018	
PA			
≤2.06 nM/L	Reference	Reference	Reference
2.07–3.21 nM/L	−268.93, 891.83, 0.76	391.05, 1476.59, 0.79	−1041.07,938.43, 0.28
3.22–5.93 nM/L	−997.02, 778.44, 0.20	784.35, 1434.70, 0.59	−2307.87, 718.25, 0.005
>5.93 nM/L	−294.59, 826.98, 0.72	983.67, 1525.03, 0.52	−1334.04, 689.14, 0.07
p for interaction		0.2046	
Hcy			
≤4.40 umol/L	Reference	Reference	Reference
4.41–5.10 umol/L	227.32, 755.24, 0.76	−692.64, 1335.38, 0.61	63.93,715.18, 0.93
5.11–6.00 umol/L	71.24,793.17, 0.93	−1992.29, 1505.14,0.20	730.08, 837.30, 0.40
>6.00 umol/L	−217.90,795.33, 0.79	−1256.44, 1479.32, 0.40	554.96, 878.78, 0.54
p for interaction		0.7041	

Adjusted for gestational age at blood draw, maternal pre-pregnancy body mass index, household income, maternal race/ethnicity, breastfeeding, and caloric intake at age 3

*BMI* body mass index, *B*_*12*_ vitamin B_12_, *PLP* pyridoxal phosphate, *PA* 4-pyridoxic acid, *Hcy* homocysteine

### Associations between maternal micronutrient concentrations and DNA methylation at DMRs regulating imprinted genes

**Fig. 1:**
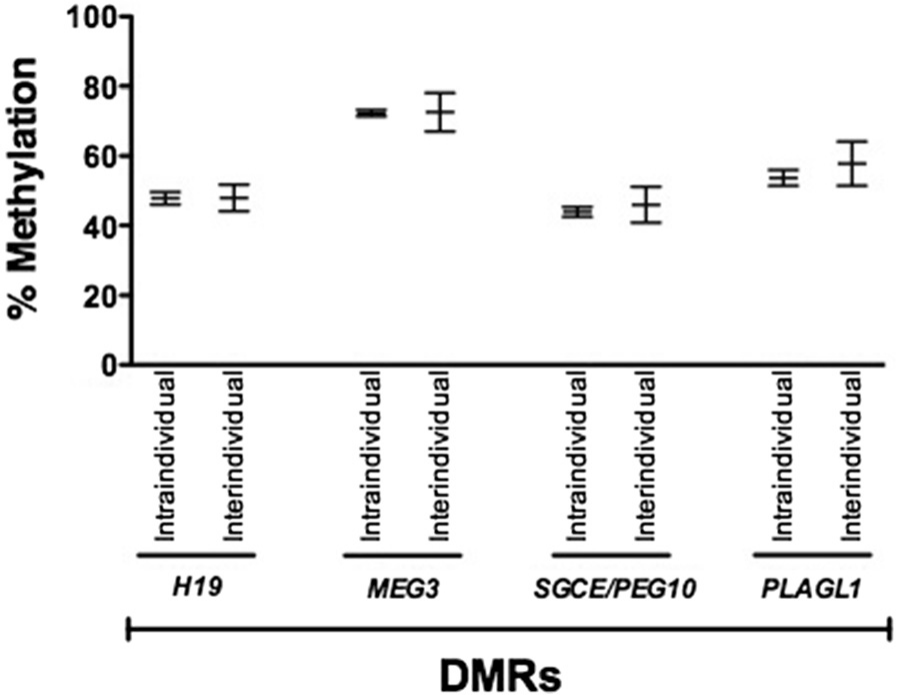
Interindividual variability exceeds intraindividual variability in DNA methylation at imprinted DMRs. Shown are the mean methylation levels, ± standard deviation for the four DMRs analyzed, alongside the means for technical replicates that were run alongside for a subset of the samples (~2 % of the total)

We examined whether maternal micronutrient concentrations were associated with imprinted DMR methylation at the *H19*, *MEG3*, *SGCE/PEG10*, and *PLAGL1* DMRs, previously shown to associate with fetal growth in our study population [17, 18]. Mean methylation % (standard deviation) for differentially methylated regions by quartile of maternal B-vitamin status are provided in Additional file 2: Table S2. After adjusting for gestational age at delivery, gestational age at blood draw, maternal race/ethnicity, maternal smoking, and pre-pregnancy BMI, we found that PLP was positively associated with methylation at the *MEG3* DMR, consistent with a threshold effect (*β*_Quartile 3_ = 2.01 and *β*_Quartile 4_ = 3.24, *p* ≤ 0.05) (Table 4). No association was found between maternal micronutrient levels and DNA methylation at other DMRs. It is possible that intraindividual variability between replicate measures could be greater than the interindividual variability across samples, which would make it difficult to decipher true differences due to exposure versus differences that occur naturally in the population. For each of the DMRs analyzed herein, the interindividual variability exceeded the intraindividual variability (Fig. 1), supporting the validity of the association identified.

**Table 4:** Adjusted regression coefficients for maternal B vitamins and infant differentially methylated regions: Newborn Epigenetic STudy (*N* = 429)

	*H19* DMR	*MEG3* DMR	*SGCE/PEG10* DMR	*PLAGL1* DMR
	mean methylation % (standard deviation)			
	47.93 (3.82)	72.64 (5.55)	45.98 (5.17)	57.79 (6.33)
Maternal B vitamins	*β* coefficient, Standard Error, *p* value			
B_12_				
≤322.47 ng/L	Reference	Reference	Reference	Reference
322.48–446.04 ng/L	0.53, 0.57, 0.35	0.53, 0.85, 0.53	0.01, 0.66, 0.98	0.39, 0.95, 0.68
446.05–575.51 ng/L	0.68, 0.56, 0.22	0.01, 0.82, 0.99	0.26, 0.66, 0.70	0.58, 0.94, 0.54
>575.51 ng/L	−0.41, 0.57, 0.48	−0.93, 0.85, 0.27	0.47, 0.67, 0.48	1.79, 0.96, 0.06
PLP				
≤3.76 nM/L	Reference	Reference	Reference	Reference
3.77–7.47 nM/L	−0.15, 0.60, 0.80	0.23, 0.83, 0.79	0.99, 0.75, 0.19	−0.02, 0.98, 0.99
7.48–12.05 nM/L	−0.68, 0.62, 0.28	2.01, 0.89, 0.03	−0.33, 0.79, 0.68	−0.26, 1.02, 0.80
>12.05 nM/L	−0.07, 0.63, 0.91	3.24, 0.89, <0.01	−0.30, 0.81,0.71	−0.11, 1.04, 0.91
PA				
≤2.06 nM/L	Reference	Reference	Reference	Reference
2.07–3.21 nM/L	−0.45, 0.58, 0.44	−0.18,0.84, 0.83	1.46, 0.74, 0.05	1.27, 0.95,0.18
3.22–5.93 nM/L	−0.09, 0.60, 0.88	−0.24, 0.87, 0.79	0.79, 0.75, 0.29	1.54, 0.98, 0.12
>5.93 nM/L	−0.57, 0.61,0.35	1.62, 0.87, 0.06	0.08, 0.78, 0.92	−0.15, 0.99, 0.88
Hcy				
≤4.40 umol/L	Reference	Reference	Reference	Reference
4.41–5.10 umol/L	1.01, 0.59, 0.09	1.49, 0.87, 0.09	1.43, 0.77, 0.06	1.46, 0.98, 0.14
5.11–6.00 umol/L	−0.97, 0.58, 0.10	1.07, 0.86, 0.21	1.19, 0.76, 0.12	1.77, 0.97, 0.07
>6.00 umol/L	0.19, 0.60, 0.75	1.60, 0.87, 0.07	1.36, 0.79, 0.09	1.10, 0.98, 0.27

Adjusted for gestational age at delivery, gestational age at blood draw, maternal race/ethnicity, maternal smoking and pre-pregnancy body mass index

*DMR* differentially methylated region, *B*_*12*_ vitamin B_12_, *PLP* pyridoxal phosphate, *PA* 4-pyridoxic acid, *Hcy* homocysteine

## Discussion

Our study did not find evidence of an association between maternal micronutrient concentrations and birth weight overall, but we observed that higher maternal Hcy concentration was associated with lower birth weight in male infants. The offspring of the mothers in the highest concentrations of B_12_ had lower WG compared to the offspring of the mothers in the lowest quartile, and higher WG was observed among male offspring of the mothers in the highest quartile of maternal PLP. Maternal PLP concentrations were positively associated with methylation at the *MEG3* DMR.

Vitamin B_12_ is essential for cellular growth and differentiation, as well as for DNA methylation, and could be an independent factor for fetal development [19]. Pregnancy-associated declines in B_12_ are common but are likely attributed to increased fetal absorption and placental transport [20]. The literature on the association between maternal vitamin B_12_ status and adverse pregnancy outcomes are mixed. A single study conducted among a cohort of pregnant women in Bangalore, India, showed that low maternal B_12_ concentrations were associated with elevated risk of intrauterine growth restriction (IUGR) [21]. However, several other investigations report no significant association between maternal B_12_ status (measured at various time points during the prenatal period) and birth weight or IUGR [7, 8, 22]. We similarly found no association with birth weight. To our knowledge, we are the first to report on the association between maternal concentrations of vitamin B_12_ and offspring WG at age 3 years. Our finding of an inverse association between maternal B_12_ concentrations and WG could suggest that maternal B vitamins during the prenatal period have downstream effects on offspring body size, and these associations may, in part, drive the inverse relationship between childhood B_12_ concentrations and obesity [23].

Plasma PLP, the best single indicator of vitamin B_6_ status, is involved in many aspects of macronutrient metabolism and is known to decline during gestation [24]. Several studies report positive associations between maternal B_6_ supplementation and birth weight, including a recent meta-analysis where a 217-g difference (95 % CI: 130–304; *p* = 0.009) was observed [24]. We observed a monotonic increase in birth weight with increasing maternal PLP concentration, but the effect was small and insignificant. We found no previous studies that examined the association between maternal PLP and offspring WG, and anthropometric data on children whose mothers received vitamin B_6_ supplements during pregnancy are not available.

The concentrations of Hcy, a sulfur-containing amino acid, are tightly regulated by two enzymatic pathways: (1) Hcy can be remethylated to methionine by a pathway requiring folate and vitamin B_12_ as a methyl donor and co-factor, respectively, or (2) Hcy may be removed by transsulfuration, a pathway reliant on vitamin B_6_ [25]. Therefore, deficiencies of folate, vitamin B_12_, or vitamin B_6_ are likely to lead to increase Hcy. Blood concentrations of Hcy during pregnancy are variable: a slight decrease during early gestation; a nadir between 20 and 32 weeks; and subsequent rise after delivery [26]. Investigations of the association between maternal total Hcy and birth weight have yielded diverging results. Many, but not all [7], studies have observed an increased risk of LBW or IUGR in women with elevated levels of total Hcy [27, 28] and a recent meta-analysis showed that hyperhomocysteinaemia (>90th percentile) was associated with a 25 % increased odds of being SGA (95 % CI: 1.09, 1.44) [29]. While we found no statistically significant associations between Hcy and birth weight overall, we did observe a novel inverse association among male infants. No previous study had evaluated WG or BMI with prenatal maternal Hcy concentrations, and our study found no significant associations.

The association between nutrients in the one-carbon pathway and offspring methylation are well-documented in animal models [30, 31], but studies among humans are limited. A cross-sectional study assessing maternal vitamin B_12_ status at the time of parturition found inverse associations with umbilical cord blood *IGF2* DMR methylation [13]. Another study showed associations between plasma levels of Hcy and cord blood DNA methylation of 289 CpG sites [32]. A recent investigation of dietary nutrients showed maternal vitamin B_2_ intake was positively correlated with *PLAGL1* DMR methylation in umbilical cord blood, although no association was found with B_6_ or B_12_ [15]. These investigations were limited by cross-sectional data collection, small samples, and inadequate assessment of maternal micronutrient status. While these associations may be by chance, our study is the first to show an association between maternal PLP and offspring methylation at the *MEG3* DMR which could be an important epigenetic tag for maternal B vitamin adequacy. Vitamin B_6_ is integrally involved in the 1-CC metabolism pathway and acts as a co-factor for epigenetic processes including DNA methylation [10]. Insufficient maternal micronutrients may affect the efficiency of the one-carbon pathway, interfering with DNA methylation and epigenetic regulation of genes such as *MEG3* during critical periods of development. *MEG3* produces a long non-coding RNA and altered expression is associated with multiple disorders including the chromosome 14 uniparental disomies [33].

Strengths of our study include its large population-based sample, prospective design, and use of blood biomarkers to assess maternal micronutrient status. We were additionally able to consider adjustment for and present results stratified by FA supplementation—although cells became small. Although we observed associations between maternal micronutrients, birth weight, childhood WG, and the *MEG3* DMR, these findings should be interpreted in context of the study limitations. While our findings could be due to chance, by considering a small number of DMRs (*N* = 4) for which we had strong biologic rationale, we (1) mitigate concerns regarding multiple comparisons; (2) reduce the likelihood of type II error; and (3) generate data which may be replicated in future studies. We assessed maternal micronutrient concentrations at a single time point and for a large proportion of women data were unavailable. Further, LBW and macrosomia were infrequent in our study population and were unable to examine associations with birth weight extremes. While we are the first to estimate associations between maternal B vitamin and Hcy concentrations and childhood WG, in some, strata sample size was reduced considerably. Finally, while there is an urgent need to better understand how maternal micronutrients involved in the 1-CC metabolism pathway affect developmental epigenetics, redundancy in methyl-donor supply pathways may indicate that alterations of one substrate could, through compensatory mechanisms, perturb others [34]. A more comprehensive approach is necessary to gain a complete understanding of how these nutrients affect DNA methylation in a larger number of regulatory regions.

## Conclusions

Our study, in a large ethnically diverse cohort of mothers and their offspring, suggests that with the exception of a sex-specific effect for Hcy, B vitamins are not associated with birth weight. However, both B_12_ and PLP appear to be associated with 3-year WG. We further showed that maternal PLP concentrations were positively associated with methylation at the *MEG3* DMR and may be important for understanding the effects of prenatal nutrition on adult health outcomes. The association between specific maternal micronutrients on the 1-CC metabolism pathway and adverse pregnancy outcomes continue to be an area of clinical and public health significance. Additional studies in large prospective birth cohorts may aid in understanding their independent and cooperative effects on fetal health, childhood outcomes, and adult disease risk. Additional mechanistic insights on the role of these nutrients and DNA methylation could provide epigenetic targets for surveillance and intervention.

## Methods

### Ethics, consent, and permissions

The study protocol was approved by the Duke University, Durham Regional Hospital and North Carolina State University Institutional Review Boards. Written, informed consent was obtained for all study participants prior to data collection.

### Study participants

The study participants were enrolled as part of the Newborn Epigenetics STudy (NEST), a prospective study of women and their offspring. Methods for enrollment of the study participants have been previously described [35]. Briefly, between 2009 and 2011, English- or Spanish-speaking pregnant women ≥18 years were identified from clinic logs of five prenatal clinics and obstetric facilities in Durham County, NC, USA. Women were excluded from the study if they did not intend to use one of the participating obstetric facilities for delivery, planned to relinquish custody of the child, move from the area in the subsequent 3 years, or had established HIV infection. Among 2548 eligible women, 1700 (66.7 %) were consented and enrolled. Women enrolled in the study were similar to those who declined with respect to age (*p* = 0.66) but dissimilar with respect to race (*p* < 0.001), where non-participating women were more likely to be Asian and Native American. Among the 1700 consenting women, 115 were excluded due to infant deaths before, during, or soon after birth. An additional 281 who were illiterate, underage, refused further participation or could not be located were excluded, such that 1304 (76.7 %) remained in the study. Of the remaining women, we measured B vitamin/Hcy concentrations from the maternal venous blood of the first 528, 63 % of which was drawn during the first trimester. These analyses are restricted to the 496 singleton infant-mother pairs in whose blood draw and birth weight data were available.

### Data collection

Data collection occurred at multiple time points throughout the study period as follows: (1) upon enrollment, participants provided peripheral blood samples (gestational age at enrollment: range = 4.0–32.5 weeks, mean = 12 weeks) and completed a self-administered questionnaire which queried women on their sociodemographic, reproductive, and lifestyle characteristics in the 6 months prior; (2) upon delivery, birth outcomes were abstracted from medical records and infant cord blood specimens were obtained to assess offspring methylation; and (3) at age 3 years, data on child anthropometry and dietary characteristics were obtained for 273 of the 496 offspring for whom maternal B vitamins were measured. The participants with and without childhood data did not differ with respect to key variables (e.g., maternal age, micronutrient concentrations, and birth weight [*p* > 0.05]).

#### Measurement of maternal micronutrient concentrations (e.g., B_12_, PLP, PA and Hcy)

Quantitative analysis of plasma B_12_ was performed using the commercial kit, ID-Vit Vitamin B_12_ (Immundiagnostic-ALPCO; Salem, NH, Ref KIF012) according to the manufacturer’s instructions. The kit uses the vitamin B_12_-dependent strain *Lactobacillus delbruekii* subsp. lactis (ATCC 7830) in a 96-well format. After processing, *L. delbruekii* growth was measured by turbidimetry at 610 nm using the Molecular Devices, Versa-Max Tunable Plate Reader. Data analysis was performed using the commercially available software Soft-Max version 3.1, Molecular Devices. PLP and PA were measured by high-performance liquid chromatography (HPLC) which requires the removal of plasma protein, conversion of the liberated PLP to 4-pyridoxic acid 5′-phosphate in alkaline medium containing cyanide (derivatization), followed by acidification. The acidified samples were subjected to reversed phase HPLC separation, and detection was carried out with fluorescence with excitation at 320 nm; emission at 416. A Thermo Separation Products System, pump model P200, autosampler model AS300, fluorescence detector model FL300 was used. Plasma Hcy was similarly assessed using HPLC with UV detection at 384 nm. Maternal micronutrient concentrations were right skewed and quartiled.

#### Assessment of birth and childhood outcomes

Trained personnel abstracted parturition data from medical records after delivery. These data included birth weight, gestational age at birth (week), and infant sex. Infant birth weight (grams [g]) showed no evidence of departure from normality and was analyzed as a continuous variable. Age 3 WG (g) was slightly right skewed and calculated using the following formula: ((age 3 weight [g]/age at which weight was obtained [months])*36 months) − birth weight [g] and assessed continuously.

#### Assessment of covariates and effect measure modifiers

The participants self-reported maternal age at delivery, race/ethnicity, marital status, parity, diabetes, and weight and height at last menstrual period (LMP), all of which were subsequently verified with abstracted medical records. Household income, maternal education, cigarette smoking, FA supplementation, and infant feeding practices were self-reported via questionnaire.

We considered maternal race/ethnicity, infant sex, maternal pre-pregnancy BMI, and FA supplementation as potential modifiers of the association between maternal micronutrient concentrations and birth weight. Race/ethnic categories were assigned based on women’s self-identification as Black/African American, non-Hispanic White, or Hispanic White. Infant sex was abstracted from medical records. Maternal BMI was calculated from self-reported weight (kg) and height (m) at LMP and expressed as kg/m^2^. FA supplementation was self-reported at baseline.

#### DNA methylation analysis

Infant genomic DNA (800 ng) was modified by treatment with sodium bisulfite using the Zymo EZ DNA Methylation kit (Zymo Research; Irvine, CA, USA). Bisulfite treatment of denatured DNA converts all unmethylated cytosines to uracils but leaves methylated cytosines unchanged, allowing quantitative definition of cytosine methylation status. Pyrosequencing was performed using Pyromark Q96 MD pyrosequencers (Qiagen) to measure DNA methylation at four imprint regulatory regions known to associate with fetal growth and development in NEST study participants [17, 18] including the following: the *H19* DMR regulating the *IGF2/H19* domain, the *MEG3* DMR regulating the *DLK1/MEG3* domain, the *SGCE/PEG10* DMR positioned between epsilon sarcoglycan and paternally expressed gene 10, and the *PLAGL1* DMR [36]. Assays were designed to query established DMRs using the Pyromark Assay Design Software (Qiagen). Polymerase chain reaction (PCR) conditions were optimized to produce a single, robust amplification product by adjusting annealing temperature and magnesium chloride concentrations. The primers, chromosomal location, and coordinates along with the PCR conditions for all four regions investigated here were previously provided [36]. Defined mixtures of fully methylated and unmethylated control DNAs were used to show a linear increase in detection of methylation values as the level of input DNA methylation increased (Pearson *r* > 0.99 for all DMRs). Once the optimal conditions were defined, each DMR was analyzed using the same amount of input DNA from each specimen (40 ng, assuming complete recovery following bisulfite modification), keeping the thermocycler and pyrosequencer constant. Controls to determine the bisulfite conversion efficiency were included for each DMR with every sample run. For all data included in the analysis, the conversion efficiency exceeded 97 %. Percent methylation for each CpG cytosine was determined using Pyro Q-CpG Software (Qiagen). We interrogated between four and eight CpG sites per DMR: four for *H19*, eight for *MEG3*, six for *SGCE/PEG10*, and six for *PLAGL1*.

### Statistical analysis

We compared the distribution of demographic and obstetric characteristics across quartiles of B_12_, PLP, PA, and Hcy using chi-squared tests [37]. Generalized linear models were used to estimate the association between maternal micronutrient concentrations (at enrollment) and birth weight as well as age 3 WG [38] (a priori two-sided *p* ≤ 0.05). For all models, we considered adjustment for the following covariates: maternal age, race/ethnicity, gestational age at delivery, marital status, parity upon enrollment, household, maternal education, maternal BMI at LMP, maternal smoking, maternal FA supplementation, gestational diabetes, gestational age at blood draw, and infant sex. For childhood WG analyses, we additionally considered adjustment for every breastfeeding, in-home smoking, height, and caloric intake at age 3 years. Final confounders were selected based on directed acyclic graphs [39] and backward elimination [40].

Among 429 term mother-infant pairs where methylation data for at least one of four DMRs was available and nutrient data were measured, we examined associations with maternal micronutrient concentrations. Study participants with DMR data available were similar to those without DMR data with respect to maternal age, micronutrient concentrations, and birth weight (*p* values >0.05). Cronbach’s alphas were >0.89 for all DMRs considered, suggesting mean methylation levels for each DMR could be used in our models [35]. We used *F* tests [40] for parametric analyses and Wilcoxon rank-sum tests [37] to examine DNA methylation levels by maternal micronutrient concentration, adjusting for factors shown to influence DNA methylation [41] (a priori *p* ≤ 0.05). All statistical analyses were conducted in SAS v9.3 (SAS Institute, Cary, NC, USA).

### Availability of Supporting Data

The data set supporting the results of this epidemiologic research will be available with appropriate human subject protection in a separate file.

## Additional files

Additional file 1: Table S1. Maternal B vitamins and infant birth weight associations by folic acid supplementation: Newborn Epigenetic Study (N = 484). Adjusted regression coefficients and standard errors for the association between maternal B vitamins (cobalamin [B_12_], pyridoxal phosphate [PLP], 4-pyridoxic acid [PA] and homocysteine [Hcy]) and infant birth weight in strata of folic acid supplementation: Newborn Epigenetic STudy. Additional file 2: Table S2. Mean methylation percentage (standard deviation) for differentially methylated regions by quartile of maternal B vitamin status. Newborn Epigenetic STudy (*N* = 429). Mean methylation percentage (standard deviation) for differentially methylated regions by quartile of maternal B vitamins (cobalamin [B_12_], pyridoxal phosphate [PLP], 4-pyridoxic acid [PA], and homocysteine [Hcy]).
