# Supplementary figures and images for: Maternal B vitamins: effects on offspring weight and DNA methylation at genomically imprinted domains
Source: Clin Epigenetics. 2016 Jan 22;8:8. doi: 10.1186/s13148-016-0174-9 (PMC4722751; doi:10.1186/s13148-016-0174-9)

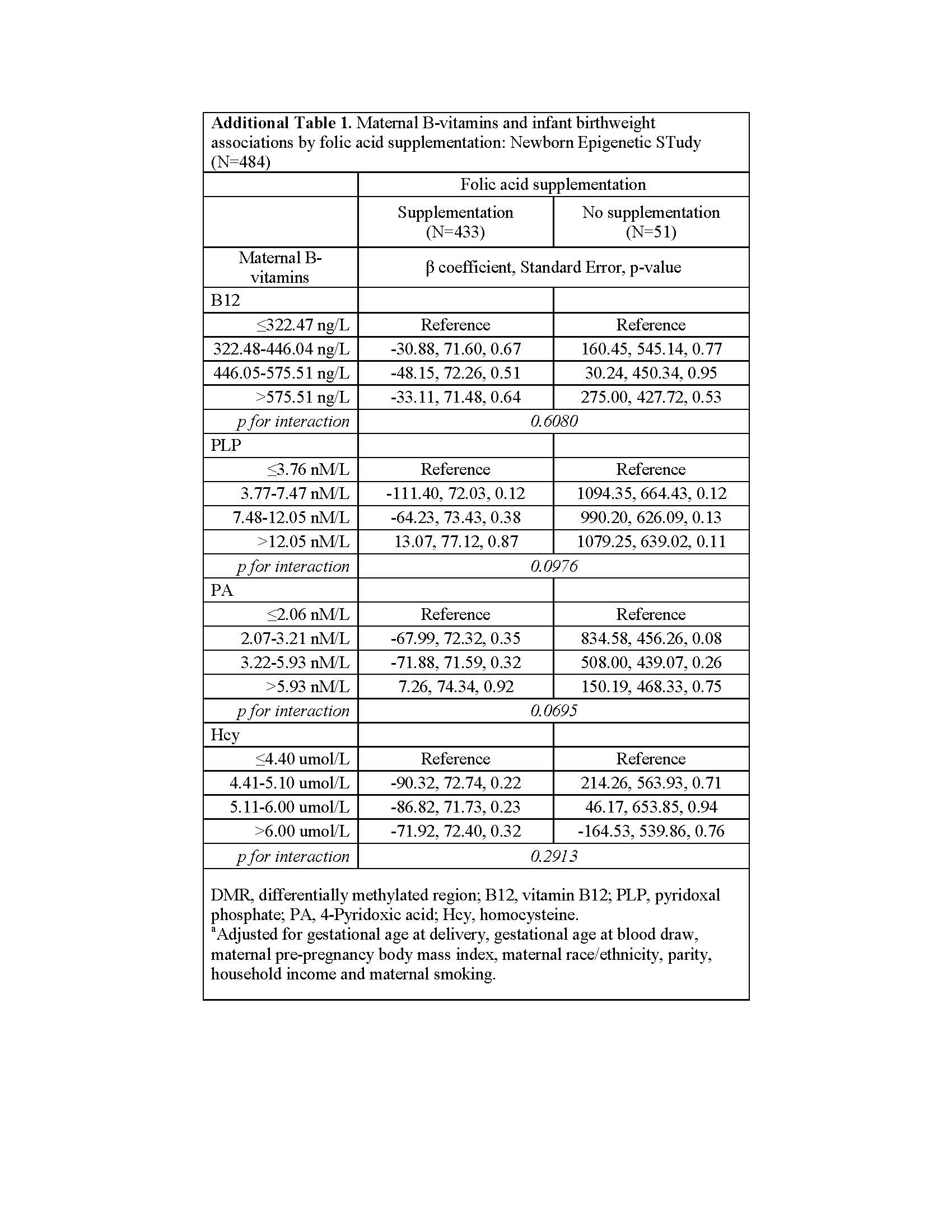

Supplement: Additional file 1: Table S1. — Maternal B vitamins and infant birth weight associations by folic acid supplementation: Newborn Epigenetic Study (N = 484). Adjusted regression coefficients and standard errors for the association between maternal B vitamins (cobalamin [B12], pyridoxal phosphate [PLP], 4-pyridoxic acid [PA] and homocysteine [Hcy]) and infant birth weight in strata of folic acid supplementation: Newborn Epigenetic STudy. [file 13148_2016_174_MOESM1_ESM.jpg]

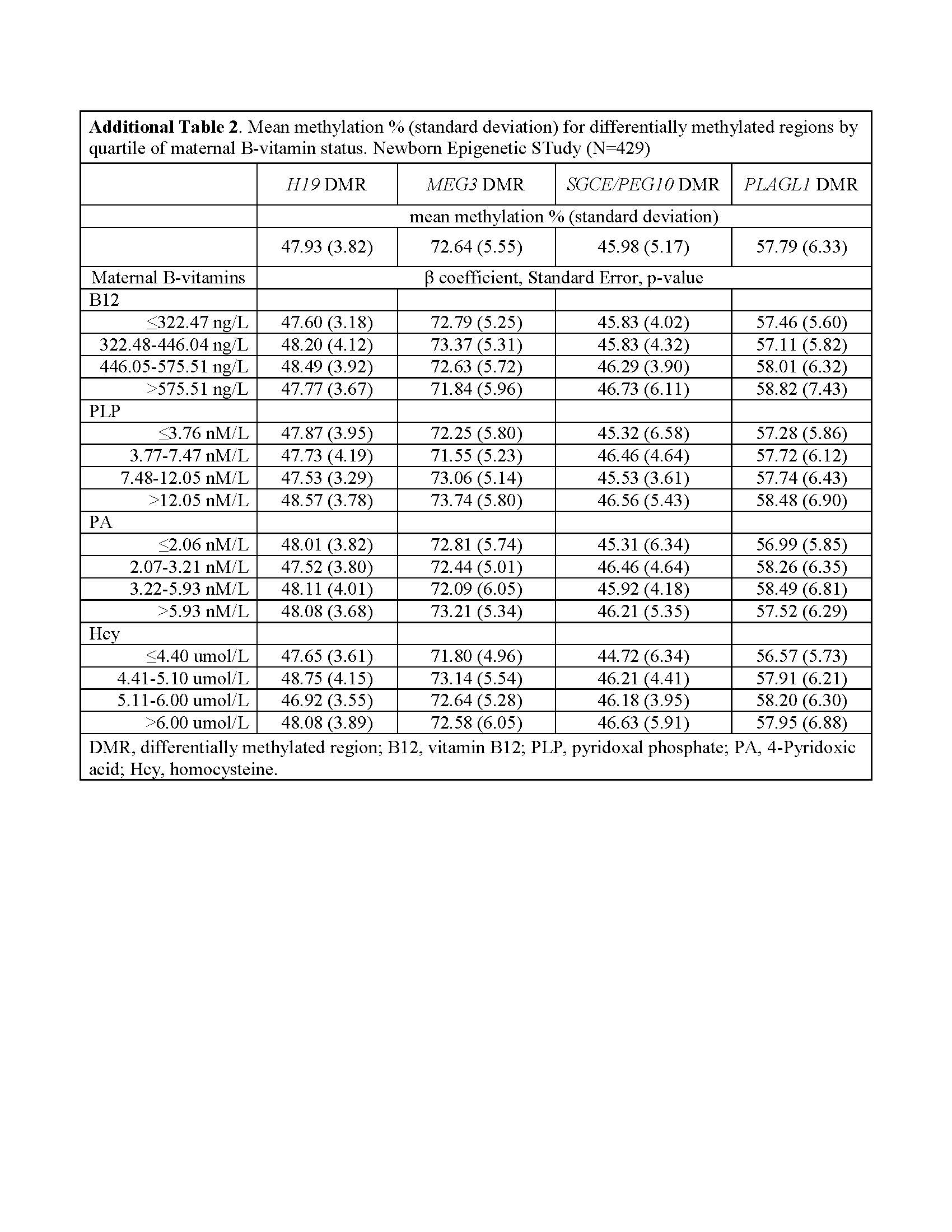

Supplement: Additional file 2: Table S2. — Mean methylation percentage (standard deviation) for differentially methylated regions by quartile of maternal B vitamin status. Newborn Epigenetic STudy (N = 429). Mean methylation percentage (standard deviation) for differentially methylated regions by quartile of maternal B vitamins (cobalamin [B12], pyridoxal phosphate [PLP], 4-pyridoxic acid [PA], and homocysteine [Hcy]). [file 13148_2016_174_MOESM2_ESM.jpg]
